# Maternal diet quality and associations with body composition and diet quality of preschool children: A longitudinal study

**DOI:** 10.1371/journal.pone.0284575

**Published:** 2023-05-11

**Authors:** Fernanda de Bona Coradi, Carolina Ribeiro Anele, Marcelo Zubaran Goldani, Clécio Homrich da Silva, Juliana Rombaldi Bernardi

**Affiliations:** 1 Graduate Program in Child and Adolescent Health, Faculty of Medical Sciences, Universidade Federal do Rio Grande do Sul (UFRGS), Porto Alegre, RS, Brazil; 2 Hospital de Clínicas de Porto Alegre (HCPA), Porto Alegre, RS, Brazil; 3 Department of Pediatrics, Faculty of Medical Sciences, Universidade Federal do Rio Grande do Sul (UFRGS), Porto Alegre, RS, Brazil; 4 Department of Nutrition, Faculty of Medical Sciences, Universidade Federal do Rio Grande do Sul (UFRGS), Porto Alegre, RS, Brazil; University of Cape Town, SOUTH AFRICA

## Abstract

**Background:**

Nutrition, associated with nutritional status, influences the growth of children. This study aimed to identify the association between maternal diet quality and the diet and body composition of their children.

**Methods:**

This is a prospective longitudinal study with mother-child pairs. To assess diet quality, nutritional status, and socioeconomic data, two interviews in the children’s first and third months of life (2011–2016) and one interview when children were of preschool age (2017–2019) were performed. Diet quality was assessed based on daily food consumption and frequency, considering: 1) food groups, based on the Brazilian food pyramid; 2) level of processing, according to the NOVA classification (unprocessed and/or minimally processed foods, processed foods and ultra-processed foods). One-way ANOVA with Tukey post hoc and Kruskal-Wallis with Dunn’s post hoc tests were used to evaluate the influence of factors on children’s diet quality. Pearson and Spearman’s correlations were used to evaluate the relationship between maternal and children’s diet quality, maternal schooling level, and child age. Along with the nutritional assessment of children, multiple linear regression models assessed the impact of covariables on maternal and children’s diet quality.

**Results:**

Eighty-three mother-child pairs participated in this study. The more frequent the maternal consumption of unprocessed and/or minimally processed foods, the higher the consumption of these foods by children (r = +0.30; p = 0.006) and the lower their subscapular skinfold (SSF) thickness (p = 0.011; β = -0.278). On the other hand, the higher the maternal consumption of ultra-processed foods, the higher the children’s tricipital skinfold (TSF) thickness (p = 0.010; β = +0.274) and SSF (p = 0.043; β = +0.222).

**Conclusion:**

Maternal diet quality was associated with the diet and body composition of children.

## Introduction

Parents play an essential role in the eating behavior of children, as their habits and lifestyle influence the children’s diet [1, 2]. Mothers are often responsible for purchasing, cooking, and supplying food for their children, which greatly affects the formation of their eating habits, body composition, and growth [1, 3, 4]. Childhood is one of the most important stages of life concerning the development of nutritional disorders and deficiencies since the quality and quantity of food are related to nutritional and health aspects [1, 5]. Consuming unhealthy foods can affect the development of short- and/or long-term health outcomes, such as childhood obesity and chronic non-communicable diseases [6–8]. Nutrition also significantly affects the growth and development of children, which are directly associated with their current and future nutritional status [8, 9]. Therefore, appropriate food consumption should be encouraged from childhood, as children are developing food habits and preferences that will last their life [2].

The relationship between maternal diet and child feeding practices and health outcomes is complex and multidimensional [3, 4]. Longitudinal studies on how maternal diet quality influences the feeding and body composition of children are necessary. In the context of anthropometric measurements, studies have shown changes in the nutritional picture of preschool children globally, with a reduction in undernutrition and an increase in childhood overweight or obesity [10, 11]. These observations have been assessed in the context of nutritional status [4, 12], child food consumption and preferences [13–17], and eating behavior [7]. However, few studies have considered maternal food preferences as an influence on the eating habits [1, 3] and body composition of children [4, 18].

This longitudinal study analyzes maternal food consumption and its influence on the diet and body composition of preschool children. Assessment of the mother-child relationship regarding food consumption can help understand the multidimensional influence of the family on the eating habits of children [3, 4].

## Methods

### Design and sample

This study was conducted in Porto Alegre, Southern Brazil, by the research group of the Center for Studies in Child and Adolescent Health of the Universidade Federal do Rio Grande do Sul (NESCA/UFRGS). Data are drawn from two prospective longitudinal studies: the Impact of Perinatal Environment Variations on the Health of the Newborn in the First Six Months of Life phase I (IVAPSA-I) and IVAPSA-II which followed up the same cohort to five years old. The study protocols [19] and baseline results were previously described [20].

IVAPSA-I (2011–2016) used a convenience sample of 400 women who had given birth to live newborns 48 to 72 hours before enrolment at three public hospitals in Porto Alegre: Hospital de Clínicas de Porto Alegre (HCPA), Hospital Nossa Senhora da Conceição, and Hospital Fêmina. They were selected from five defined groups of pregnant women with different clinical gestational conditions: smokers, women with diabetes, hypertension, or intrauterine growth restriction (IUGR) (i.e., small-for-gestational-age newborns), and a control group, without any of the previous conditions. Each group had only one gestational clinical condition. Twin or preterm newborns, newborns with congenital malformations or some infectious diseases, and those who required early hospitalization were excluded, based on the exclusion criteria of the IVAPSA. Interviews were performed in the children’s first and third months of life. In total, 223 mother-child pairs remained in the study (55.7% of the initial sample) at the end of IVAPSA I sixth months old.

IVAPSA-II (2017–2019) enrolled the same cohort and included a single interview when children were aged three to six years old. 128 (32% of IVAPSA-I) mother-child pairs were included. This sample reduction occurred due to incidental factors, such as municipality and change of contact phone number, which made it difficult to locate individuals and/or made it impossible for them to participate on the scheduled collection days, or because individuals refused to undergo follow-up, among other factors.

**Fig 1:**
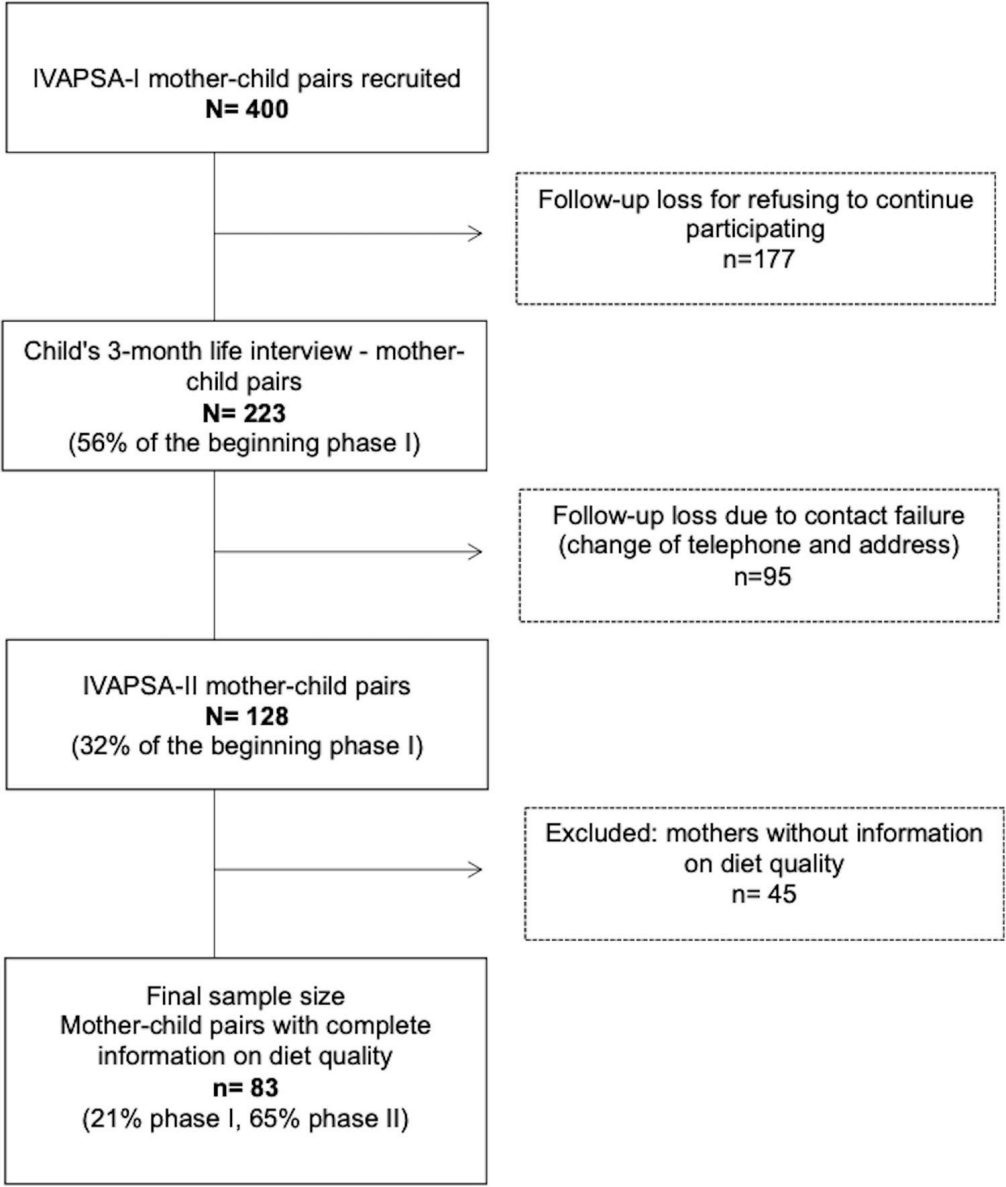
Flow chart of participants in study.

Mother-child pairs without information on diet quality were excluded from this study and a total of 83 pairs with complete information on food consumption were analyzed (Fig 1). In our study, different maternal clinical conditions and different intrauterine environments did not associated with the body composition of preschool children. In this way, these different groups were analyzed in combination.

Included and excluded mother-child pairs were compared by maternal age, household income, maternal body mass index (BMI), marital status, maternal schooling level, number of children, age of children, attendance in preschool, children’s BMI/A, duration of breastfeeding, and use of infant formula, and showed no statistically significant difference (p>0.05).

The power calculation was performed in Winpepi™ version 11.25 based on a study by Groele and collaborators, considering the correlation of fruit consumption between mothers and children [16], with r_S_ = 0.4, a power of 95%, and a significance level of 5%. Thus, the minimum sample size for the two-tailed test was 76 mother-child pairs. The original study protocol, the sample calculation, and its preliminary results were previously published [19].

### Data collection

The interviewers were trained by nutritionists and the healthcare team conducted all stages of the study to reduce bias and standardize the collection. Interviews in the children’s first and third months of life (2011–2016) were performed in the house of mother-child pairs and the children’s food consumption, including breastfeeding and the use of infant formula, was evaluated by the 24-hour dietary recall (24hDR) and maternal anamnesis.

Interviews occurred when children aged three to six years (2017–2019) and were performed in the HCPA Clinical Research Center (CPC). Urban transport tickets were made available for mothers and children. For this interview, the 24hDR, a food frequency questionnaire (FFQ) (S1 File), a sociodemographic questionnaire, and anthropometric measurements were used. A questionnaire assessed the time of exposure of children to television and smartphones.

### Diet quality

Diet quality was assessed based on the daily consumption of a given food group (Brazilian food pyramid) or processing category and food frequency. By the 24hDR, mothers reported their food and beverage consumption from midnight of the previous day to midnight of the day of the interview. A photographic album of portions and measurements [21] was used to help mothers report. To minimize the potential effect of diet variation during the three interviews, the three 24hDR were grouped. Interviews were conducted from Mondays to Saturdays, respecting the availability of mothers, thus, the 24hDR shows food consumption on weekdays and weekends, without establishing a random pattern due to this availability.

**Fig 2:**
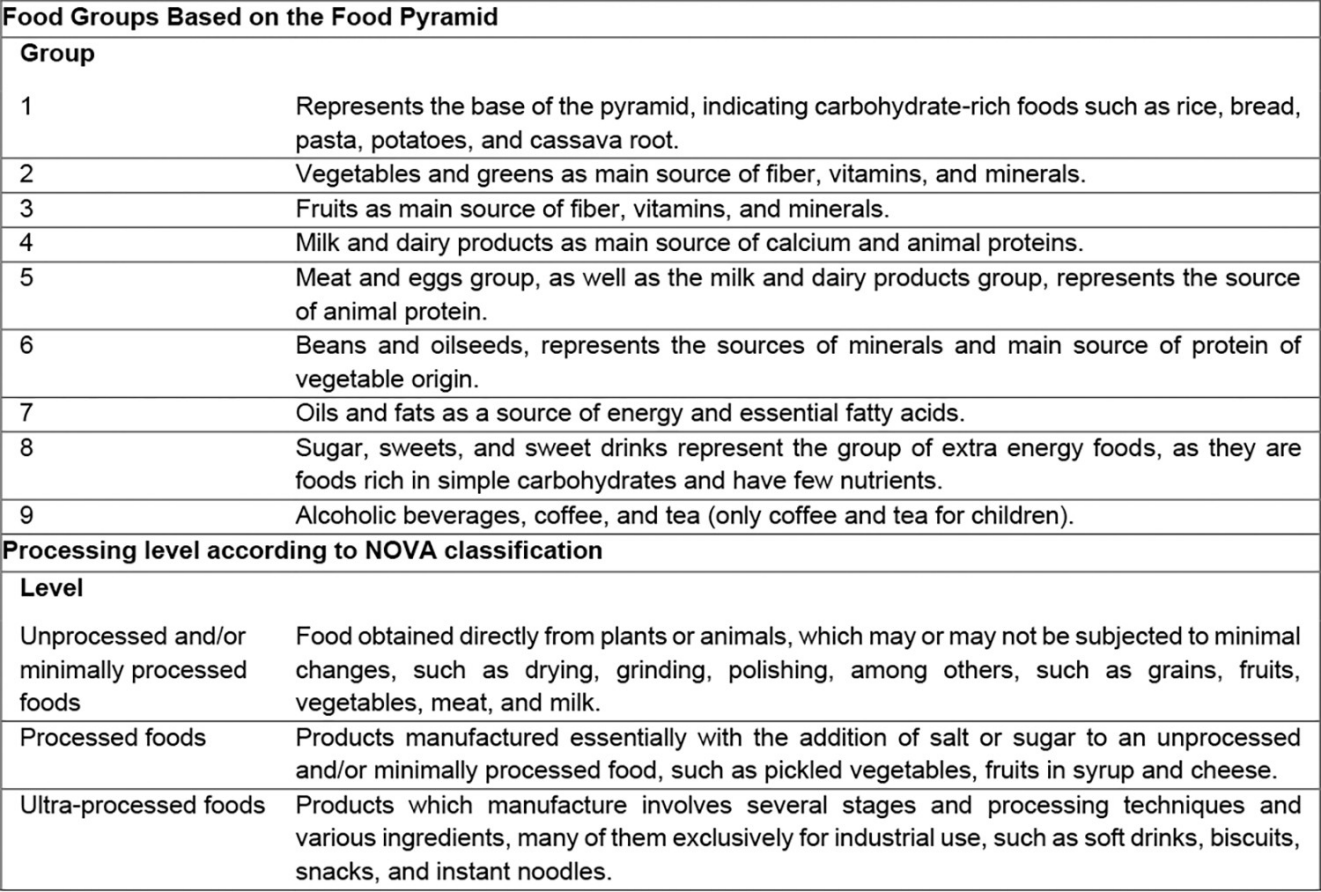
Classification of food groups based on the Brazilian food pyramid adapted for this study and processing levels according to the NOVA classification system. Source: adapted from Philippi ST (2014); Monteiro (2010, 2016).

The FFQ for children was completed by their mothers. Oral instructions were given for its correct completion and the interviewers clarified doubts. This questionnaire was previously validated for children aged two to five years [22]. The FFQ includes for each food simple questions with multiple and closed answers, divided into seven categories: never, less than once a month, one to three times a month, once a week, two to four times a week, once a day, and two or more times a day. All questions had the same options (Food Frequency Questionnaire–S1 File). The answers of both the 24hDR and the FFQ were converted into the frequency of daily consumption, considering the number of times a specific food was consumed, and later classified according to 1) food groups, based on the Brazilian food pyramid [23]; 2) level of processing, according to the NOVA classification (unprocessed and/or minimally processed foods, processed foods, and ultra-processed foods) [24, 25], when not associated with a culinary preparation or processed food, for example, adding sugar to tea. Finally, based on the classification, the daily frequency and proportion of a given food group or processing category were determined. The food pyramid was adapted, with the inclusion of group 9—alcoholic beverages, coffee, and tea (only coffee and tea for children)—to separately evaluate these foods, which were frequently consumed in the study population. Regarding the classification by processing level according to NOVA, as the frequency of these foods was lower and these foods were already separately in group 7 in the food pyramid, in this study, “processed culinary ingredients” was included in the same level as “unprocessed and/or minimally processed foods” (Fig 2 NOVA Classification).

### Assessment of nutritional status

Anthropometric measurements were evaluated in duplicate and performed using standard techniques and calibrated equipment [26]. For body weight (kg), a portable digital electronic scale (Marte™) with an accuracy of 50 grams was used and the mother-child pairs were weighed with the minimum of clothing. For height (cm), an extensible portable stadiometer (Alturexata™ Belo Horizonte, Brazil) was used. The tricipital skinfold (TSF) (mm) and subscapular skinfold (SSF) (mm) of children were measured using the Lange™ adipometer.

The maternal nutritional status through the BMI was classified according to the World Health Organization (WHO) classification [21]. The nutritional status of children was classified according to the z-score value of the WHO growth curve [27] in the WHO Anthro™ and WHO Anthro Plus™ software for Windows, by sex and age group, resulting in the classification of body mass index for age (BMI/A) as thinness, normal weight, risk of overweight, overweight, obesity, and severe obesity. However, due to the sample size, which would make each group have few individuals, classifications were grouped into three categories: normal and risk of overweight, overweight, and obesity. For the classification and evaluation of TSF and SSF, the percentile of Frisancho scales [28] was used, considering excess adiposity in children with 90% or more in TSF and SSF.

### Covariates

Socioeconomic data, including the age of mothers and children (complete years), sex of the children (girl or boy), maternal schooling level (years of study), number of children, marital status (married or living together, or single), receipt of any government benefit, and household income (≤1 minimum wage [MW], 1–3 MW or ≥3 MW) were collected. The MW during the study period was R$ 1,100.00 [29]. The time of exposure of children to television and smartphones was evaluated in minutes per day and categorized into three groups: <120 minutes, 120–240 minutes, and ≥240 minutes [30]. Only one child followed the American Academy of Pediatrics (AAP) [31] recommendation of using screens for up to 60 minutes/per day.

### Statistical analysis

A descriptive statistical analysis of socioeconomic data, diet quality, and nutritional status was performed. It presented absolute and relative frequencies (for categorical variables), means and standard deviation (for continuous parametric distributed variables), and medians and interquartile range (for continuous nonparametric distributed variables).

To assess the normality condition of variables, the Shapiro-Wilk test, with a significance level of 95% and p<0.05, was used. One-way ANOVA with Tukey post hoc (parametric data) and Kruskal-Wallis post hoc with the Dunn test (nonparametric data) was used to evaluate the relationship between cofactors (household income and time of exposure to television and smartphones) on children’s diet quality. Pearson (parametric data) and Spearman correlations (nonparametric data) were used to assessing the relationship between children’s diet quality and socioeconomic factors (maternal schooling level and age of children) and the relationship between maternal and children’s diet quality. Finally, by the Pearson test, the correlation between maternal and children’s diet quality (according to the level of processing) and the nutritional status of children was evaluated.

Four multiple linear regression models were built and adjusted for variables with statistical significance by the bivariate test (p<0.20). In model 1, the association between maternal diet and the nutritional assessment of children (TSF and SSF), adjusted for household income and maternal schooling level, was assessed. In model 2, the association between child feeding and BMI/A, adjusted for time of exposure to television and smartphones and maternal schooling level, was assessed. In model 3, the association between child diet and TSF, adjusted for time of exposure to television and smartphones, maternal schooling level, and household income was assessed. In model 4, the association between child diet and SSF, adjusted for time of exposure to television and smartphones, maternal schooling level, household income, and the age of children, was assessed.

Analyses were performed in the Statistical Package for the Social Sciences (SPSS)® version 18.0, with double entry, and later transferred to Epi Info® version 6.0, to validate duplicates.

### Ethical aspects

In both phases of the study, the mothers signed an informed consent form for their participation in the study as well as for their children. This study was approved by the HCPA Ethics Committee, under no. 11–0097 in the first phase and no. 17–0107 in the second phase (CAE 65190217500005327), and the GHC, under no. 11–027.

## Results

This study analyzed 83 mother-child pairs with complete information on food consumption from 2017 to 2019. Table 1 shows the sample characteristics. The mean maternal age was 32.81±7.11 years old, the most prevalent household income was between one and three MW (n = 52; 62.7%), and most women were overweight (n = 58; 69.9%), married or living together (n = 56; 67.5%), had two children, and studied for 10.5±3.5 years. Most children were girls (n = 49; 59%), breastfed for more than 12 months (n = 43; 51.8%), and did not use infant formula (n = 46; 55.4%). According to BMI/A, 16.9% (n = 14) of children were overweight and 13.2% (n = 11) had obesity. Regarding skinfold measurements, 24% (n = 20), according to TSF, and 34.6% (n = 28), according to SSF, had excess adiposity and no children had low adiposity.

**Table 1:** Characteristics of mother-child pairs (n = 83) from 2017 to 2019.

	Characteristic	
	**Age (years) (mean±SD)**	32.81±7.11
Mother	**Schooling level (years) (mean±SD)**	10.5±3.5
	**Household Income with benefits n (%)** ^*^	
	≤1 minimum wage	20 (21.1)
	1–3 minimum wages	52 (62.7)
	≥3 minimum wages	11 (13.3)
	**BMI (kg/m^2^) n (%)**	
	Adequate	25 (30.1)
	Overweight	58 (69.9)
	**Number of children median (IQR)**	2 (1–2)
	**Marital status n (%)**	
	Married/living together	56 (67.5)
	Single	28 (32.5)
	**Intrauterine environment n (%)**	
	Control	38 (45.8)
	Diabetes	14 (16.9)
	Hypertension	12 (14.5)
	Smoker	12 (14.5)
	SGA	7 (8.4)
Child	**Age (years) mean (±SD)**	4.58±0.78
	**Sex**	
	Girl	49 (59.0)
	Boy	34 (41.0)
	**Time of exposure to television and smartphones (minutes/day) n (%)**	
	<120	17 (20.5)
	120–240	50 (60.2)
	≥240	16 (19.3)
	**BMI/A (kg/m^2^) n (%)**	
	Normal and risk of overweight	58 (69.9)
	Overweight	14 (16.9)
	Obesity and severe obesity	11 (13.2)
	**TSF (mm) n (%)**	
	Adequate	63 (76.0)
	Excess adiposity	20 (24.0)
	**SSF (mm) n (%)**	
	Adequate	53 (65.4)
	Excess adiposity	28 (34.6)
	**Duration of breastfeeding n (%)**	
	Never	2 (2.4)
	<6 months	20 (24.1)
	6–12 months	18 (21.7)
	>12 months	43 (51.8)
	**Use of infant formula n (%)**	
	Not receive	46 (55.4)
	<6 months	27 (32.6)
	6–12 months	8 (9.6)
	>12 months	2 (2.4)

n: number of observations. %: percentage. <: less than. ≥: greater than or equal to. >: greater than. SD: standard deviation. BMI: body mass index. TSF: tricipital skinfold. SSF: subscapular skinfold. IQR: interquartile range. SGA: small for gestational age; BMI/A: body mass index for age.

*Current minimum wage: R$ 1,100.00

Table 2 presents the descriptive analysis and SD of maternal and children’s food consumption by food group (Brazilian food pyramid) and level of processing (NOVA).

**Table 2:** Mean daily consumption of Brazilian food pyramid groups (food frequency) of mother-child pairs according to the level of processing (NOVA).

	Unprocessed and/or minimally processed foods		Processed foods		Ultra-processed foods	
	Mother	Child	Mother	Child	Mother	Child
**Group 1**	42.9±15.1	39.2±14.6	27.5±13	27.7±12.4	29.6±18.1	33.1±14.3
**Group 2**	80.1±26.0	73.8±25.2	10.5±20.6	26.2±25.2	9.4±15.1	-
**Group 3**	99.5±3.4	100±0	0.5±3.4	-	-	-
**Group 4**	51.4±35.2	77.6±19.5	35.8±33.6	-	12.8±21	22.4±19.5
**Group 5**	63.9±21.3	40.5±24.6	8.9±13.1	23.3±22.2	27.2±19.7	36.2±20.4
**Group 6**	95.7±12.5	100±0	3.4±9.9	-	0.9±5.6	-
**Group 7**	67.4±17.1	100±0	-	-	32.6±17.1	-
**Group 8**	6.4±12.9	-	7.5±15.3	-	86.1 ±19	100±0
**Group 9**	95.7±13.9	57.6±43.7	0.9±6.1	42.4±43.7	3.4±12.8	-

Data presented in mean and standard deviation. SD: standard deviation. -: no consumption of these foods.

We also evaluated some of the social, economic, and environmental factors involved in the mother-child feeding relationship. Maternal consumption according to food group and level of processing was statistically significantly associated with household income and maternal schooling level. Mothers with a higher schooling level consumed more group 2 (vegetables and greens) (r = +0.216; p = 0.050) and group 4 foods (milk and dairy products) (r = +0.248; p = 0.024) and less group 7 foods (oil and fats) (r = −341; p = 0.002) (S1 Table).

Regarding children’s diet quality, there were significant differences in the average frequency of food consumption according to household income and time of exposure of children to television and smartphones. When mothers have a higher schooling level, more group 6 foods (beans and oilseeds) (r = +0.443; p<0.001) and less group 2 (vegetables and greens) (r = −0.286; p = 0.009) and group 9 foods (coffee and tea) (r = −0.358; p = 0.001) children consume. Regarding age, younger children consume more group 6 (beans and oilseeds) (r = −0.244; p = 0.026) and unprocessed and/or minimally processed foods (r = −0.224; p = 0.041).

The consumption of unprocessed and/or minimally processed foods was higher among children with household incomes higher than or equal to three MW when compared with incomes of one to three MW (p = 0.033). The consumption of ultra-processed foods was higher among children with household incomes of one to three MW (p = 0.014) when compared with monthly incomes higher than or equal to three MW (Table 3). Children whose time of exposure to screens was longer than or equal to 240 minutes (p = 0.007) presented the highest consumption of processed foods compared with less than 120 minutes/day (Table 3).

**Table 3:** Associations between sociodemographic characteristics and children’s diet quality.

	Household income (MW)*			Time of exposure to television and smartphones (minutes/day)*			Maternal schooling level (years)**			Child’s age (years old)**		
**Food group**	≤1	1–3	≥3	<120	120–240	≥240	**p**	**r**	**p**	**p**	**r**	**p**
Group 1	24.5±4.3	26.1±7.7	23.3±5.0	24.5±5.4	26.0±7.3	24.0±6.2	0.383	−0.106	0.341	0.528	0.082	0.459
Group 2	8.8±6.0	10.4±6.1	11.8±9.6	9.6±5.3	10.0±7.4	11.2±5.5	0.457	−0.286	0.009****	0.771	0.118	0.288
Group 3	8.5 (4.9–12.3)	8.1 (4.7–12.0)	13.1 (7.6–16.7)	9.8 (6.9–12.5)	8.3 (4.3–13.6)	7.7 (5.1–11.8)	0.057	−0.024***	0.830	0.898	−0.091***	0.411
Group 4	13.4±6.7	12.1±4.9	8.6±4.7	12.2±4.5	11.9±5.6	11.9±6.3	0.061	0.176	0.112	0.980	−0.131	0.238
Group 5	9.5±5.6	9.4±5.5	7.7±5.3	7.8±5.4	9.1±5.1	10.8±6.3	0.465	0.209	0.058	0.302	0.207	0.060
Group 6	11.7±5.2	10.5±4.7	12.5±8.1	9.7±4.9	11.6±5.5	10.9±5.0	0.695	0.443	0.001****	0.432	−0.244	0.026****
Group 7	4.4±3.4	4.0±2.7	2.5±2.7	3.3±2.1	4.3±3.12	3.3±2.6	0.203	0.077	0.489	0.309	−0.052	0.641
Group 8	17.6±7.4	18.0±6.7	16.5±8.5	20.4±7.7	16.7±6.7	18.1±7.2	0.801	−0.181	0.102	0.164	0.069	0.533
Group 9	0.0 (0.0–1.4)	0.0 (0.0–0.54)	0.28 (0.0–1.19)	0.4 (0.0–2.1)	0.0 (0.0–0.4)	0.18 (0.0–1.1)	0.380	−0.358***	0.001****	0.276	0.101***	0.366
**Level of processing**	≤1	1–3	≥3	<120	120–240	≥240	**p**	**r**	**p**	**p**	**r**	**p**
Unprocessed and/or minimally processed food	53.4±10.8^ab^	49.8±10.7^a^	59.1±11.7^b^	52.3±14.0	52.5±10.8	49.5±9.2	0.033	0.052	0.642	0.638	−0.224	0.041****
Processed food	14.0±6.2	12.8±5.4	12.9±6.6	10.2±4.4^a^	13.0±5.2^ab^	16.4±6.9^b^	0.723	−0.131	0.238	0.007	0.125	0.261
Ultra-processed food	29.5±10.5^ab^	34.3±9.1^b^	26.2±6.9^a^	32.2±12.4	32.1±8.6	32.2±9.9	0.014	−0.231	0.036****	0.999	0.203	0.065

p: p-value. MW: minimum wage (1 MW = R$ 1,100.00). Significance level = 0.05. Data with normal distribution were presented as mean±SD and for variables with asymmetric distribution, they were presented as median (interquartile range). *Mean±SD–ANOVA, Tukey post hoc. Median (P25–P75). Kruskal-Wallis test.

^ab^ Different letters represent significant differences between one-way ANOVA and the Tukey test.

**Pearson correlation. ***Spearman correlation. ****Significant correlation.

Table 4 shows the proportions of maternal consumption of unprocessed and/or minimally processed, processed, and ultra-processed foods were 58.5%, 12.9%, and 28.7%, respectively. For children, these proportions were 51.9%, 13.1%, and 32.1%, respectively. When comparing maternal and children’s food consumption, we found a positive correlation and a statistically significant result regarding the consumption of unprocessed and/or minimally processed foods (r = +0.30; p = 0.006).

**Table 4:** Relationship between maternal and children’s diet quality according to the level of food processing.

Food group	Mother		Child		
	Mean±SD	Median (IQR)	Mean±SD	Median (IQR)	r (p)*
Group 1	22.8±4.74	22.6 (19.5–25.5)	25.3±6.76	25.3 (20.6–29.3)	0.14 (0.187)
Group 2	13.2±6.94	13.3 (7.7–16.7)	10.2±6.62	9.4 (5.4–13.5)	0.11 (0304)
Group 3	3.6±3.97	2.6 (0.0–6.3)	9.3±6.08	8.6 (5.1–12.8)	0.08 (0.464)**
Group 4	8.1±4.09	8.1 (4.8–10.3)	12±5.51	11.9 (7.8–14.7)	0.13 (0.235)
Group 5	14.1±3.91	14.3 (11.3–16.7)	9.2±5.47	8.7 (4.1–12.5)	0.22 (0.049)
Group 6	6.0±3.09	6.5 (4.1–7.5)	11.1±5.32	10.9 (7.2–14.4)	0.17 (0.121)
Group 7	13.3±3.38	13.8 (11.1–15.1)	3.9±2.87	3.8 (1.7–5.6)	0.13 (0.262)
Group 8	12.9±5.93	11.1 (8.2–15.8)	17.7±7.08	17.5 (13.2–22.5)	0.12 (0.274)
Group 9	6.5±4.13	6.3 (3.4–10.2)	1.3±2.84	0.0 (0.0–0.6)	0.11 (0.318)**
**Level of processing**					
Unprocessed and/or minimally processed food	58.5±11.12	60.0 (51–66.7)	51.9±11.18	52.3 (43.8–59.2)	0.30 (0.006)***
Processed food	12.9±5.14	12.2 (9.3–16.2)	13.1±5.71	12.6 (9.1–12.3)	0.12 (0.289)
Ultra-processed food	28.7±10.04	26.9 (22.2–35.1)	32.1±9.62	31.0 (24.6–38.7)	0.14 (0.218)

SD: standard deviation. IQR: interquartile range

*Pearson correlation coefficient, with a significance level of 0.05.

**Spearman correlation coefficient, with a significance level of 0.05.

***Statistically significant result.

There was found no statistically significant difference between the maternal and children’s consumption of food groups and child anthropometry (p>0.05). Maternal and children’s diet quality by level of food processing was correlated with child anthropometry. When correlating maternal diet quality with child anthropometry, the higher the maternal consumption of unprocessed and/or minimally processed foods, the lower children’s SSF (adjusted analysis; p = 0.011; β = −0.278), and when the higher the maternal consumption of ultra-processed foods, the higher children’s TSF (adjusted analysis; p = 0.010; β = +0.274) and SSF (adjusted analysis; p = 0.043; β = +0.222) (Table 5). Regarding children’s diet quality, higher consumption of processed foods was associated with higher TSF (adjusted analysis; p = 0.001; β = +0.347) and SSF (adjusted analysis; p = 0.013; β = +0.283) (Table 6).

**Table 5:** Association between maternal diet quality by the level of food processing on the nutritional status of children (model 1).

Level of processing	BMI/A					TSF					SSF				
Mother feeding^‖^															
Unprocessed and/or minimally processed food	−0.09(0.436)	-0.017 (-0.04–0.00)	-0.135	0.201	0.156	−0.19(0.092)	−0.474 (−0.95–0.00)	−0.211	0.049***	0.128	−0.27(0.014)***	−0.649 (−1.140–−016)	−0.278	0.011***	0.144
Processed food	−0.07(0.557)	0.000	-0.002	0.989	0.138	−0.11(0.322)	-0.387 (-1,44–0.67)	-0.080	0.470	0.079	0.13(0.231)	0.898 (−0.23–2.02)	0.173	0.117	0.097
Ultra-processed food	0.13(0.244)	0.020	0.148	0.156	0.160	0.26(0.016)***	0.683 (0.17–0.20)	0.274	0.010***	0.158	0.23(0.036)***	0.575 (−0.02–−1.13)	0.222	0.043***	0.116

BMI/A: body mass index for age. TSF: tricipital skinfold. SSF: subscapular skinfold.

*R: Pearson correlation coefficient, with a significance level of 0.05.

**Multiple linear regression p = 0.05. b: unstandardized coefficient. β: regression coefficient. 95%CI: 95% confidence interval. R^2:^ R squared.

***Statistically significant result.

Multiple linear regression models were built and adjusted for variables with statistical significance (p<0.20 in the Pearson correlation).

^‖^Model 1: Maternal feeding on the nutritional status of children (BMI/A, TSF, and SSF), adjusted for household income and maternal schooling level.

**Table 6:** Association between children’s diet quality by the level of food processing on the nutritional status of children (models 2, 3 and 4).

Level of processing	BMI/A^┼^					TSF^⌂^					SSF^◊^				
Child feeding^┼,⌂,◊^															
	r(p)*	b (95%CI)	β	p**	R^2^	r(p)*	b (95%CI)	β	p**	R^2^	r(p)*	b (95%CI)	β	p**	R^2^
Unprocessed and/or minimally processed food	−0.10(0.364)	-0.007 (-0,03–0,02)	-0.053	0.637	0.053	−0.02(0.864)	0.076 (-0.42–0.57)	0.034	0.761	0.093	−0.14(0.206)	−0.289 (−0.80–0.22)	−0.125	0.266	0.114
Processed food	0.21(0.052)	0.052 (0.00–0.10)	0.214	0.058	0.093	0.34(0.001)***	1.516 (0.60–2.43)	0.347	0.001***	0.192	0.30(0.006)***	1.306 (0.28–2.33)	0.283	0.013***	0.112
Ultra-processed food	−0.09 (0.936)	0.009 (-0.04–0.02)	-0.064	0.572	0.054	−0.12(0.279)	−0.445 (−1.01–0.12)	−0.171	0.122	0.112	0.03 (0.828)	-0.068 (-0.68–0.54)	0.025	0.827	0.117

BMI/A: body mass index for age. TSF: tricipital skinfold. SSF: subscapular skinfold.

*R: Pearson correlation coefficient, with a significance level of 0.05.

**Multiple linear regression p = 0.05. b: unstandardized coefficient. β: regression coefficient. 95%CI: 95% confidence interval. R^2:^ R squared.

***Statistically significant result.

Multiple linear regression models were built and adjusted for variables with statistical significance (p<0.20 in the Pearson correlation).

^┼^Model 2: Child feeding on BMI/A, adjusted for time of exposure to screens and maternal schooling level.

^⌂^Model 3: Child feeding on TSF, adjusted for time of exposure to screens, maternal schooling level, and household income.

^◊^Model 4: Child feeding on SSF, adjusted for time of exposure to screens, maternal schooling level, household income, and the age of children.

## Discussion

In this study, maternal diet quality was associated with children’s diet quality and TSF and SSF thickness. The more frequent the maternal consumption of unprocessed and/or minimally processed foods, the more frequent the consumption of these foods by children and the lower values of their SSF. Excess adiposity by TSF and SSF in children was correlated with higher consumption of processed foods by children and ultra-processed foods by mothers. Moreover, children’s diet quality was associated with their age and maternal schooling level.

We found significant differences according to time of exposure to television and smartphones and household income, showing that the family environment plays a central role in the feeding patterns and nutritional status of preschool children.

Maternal schooling level and family income have a positive effect on children’s diets with more essential foods such as beans and oilseeds consumed at higher levels of income and education, but also with lower consumption of vegetables and greens. Several studies show the relationship between maternal schooling level [13, 32–37] and household income in child feeding [16, 33–35, 38]. Individuals with higher schooling levels tend to choose healthier foods and have greater access to healthy and nutritional information, especially about which healthy foods to give to their children [32, 39, 40]. In this study, the consumption of vegetables and greens (group 2) was an exception, but this may represent the small sample size, which in general shows a low consumption of these foods even in families with higher purchasing power [41].

Higher household incomes were related to higher schooling levels and, consequently, more information about healthy eating and access to food, besides the cost of purchasing unprocessed foods. Batalha et al. (2017) [32] and Drouillet-Pinard et al. (2017) [34] showed that higher consumption of ultra-processed foods by children was associated with a lower maternal schooling level. On the other hand, Linhares et al. (2020) [37], in a study with Brazilian children aged one to six years, found that a lower maternal schooling level was associated with higher consumption of beans by children. Claro et al. (2016) [41] showed that some unprocessed foods tend to cost more than ultra-processed foods and families with higher purchasing power tend to pay more for their purchases compared with families with lower purchasing power. Corroborating the literature, this study showed that a higher household income was related to higher consumption of unprocessed and/or minimally processed foods, and a lower income was associated with higher consumption of ultra-processed foods.

This study showed the relationship between the consumption of processed foods by children and a greater time of exposure to screens, which may be related to the exposure to unhealthy food advertisements while watching shows, convenience in purchasing, and having ready-to-eat meals. Similarly to our results, studies found that a longer screen time was associated with higher consumption of unhealthy foods by children [39, 42]. AAP recommends limiting the exposure of children aged three to five years to digital devices to a maximum of 60 minutes per day, always with the supervision of a parent or guardian, and restricting access to television and smartphones during meals, aiming to avoid digital adversities and prevent health outcomes [31]. Low exposure to television and smartphones, as well as changing children’s eating habits are appropriate strategies to reduce the consumption of processed and ultra-processed foods [38, 42]. Based on these findings, studies on this topic are even more necessary to clarify the relationship between food consumption and exposure to digital devices in preschool children using the NOVA classification.

According to our findings, younger children consume healthier foods, such as beans and oilseeds (group 6), and unprocessed and/or minimally processed foods more often. The lower frequency of consumption of unprocessed and/or minimally processed foods by older children may be related to the beginning of independence, as when they are younger, adults are primarily responsible for choosing their food, and over time children develop autonomy, even if they depend on those responsible for the purchase, and their choices are influenced by environmental, cultural, and social factors, including family eating habits and media and advertising [1, 17, 43–45]. Linhares et al. (2020) [37] found higher consumption of fruits and vegetables among children aged up to two years compared with those aged three and four. Healthy eating must be encouraged through nutritional education and access to food since before children’s food introduction, and when seeking healthy eating for children, families should do the same for them.

In most families, mothers are responsible for buying and supplying food, as well as preparing meals [4, 14–16, 46], which is one of the hypotheses as to why their habits can influence children’s diet. This study assessed the relationship between maternal and children’s diet quality, in which the maternal frequency of consumption of unprocessed and/or minimally processed foods was correlated with a higher frequency of consumption of these foods. Other authors have investigated the influence of maternal diet quality on their children [14–17]. Groele et al. (2018) [16] analyzed mothers and preschool children in Romania and Poland and found that the maternal preference for fruits was decisive in their consumption by children. These authors also evaluated the effect of maternal preferences on the consumption of vegetables and found a connection between preferences in the consumption of some vegetables according to both quantity and variety of food [14].

Maternal and children’s diet quality influenced child anthropometry. Higher maternal consumption of unprocessed and/or minimally processed foods was correlated with a reduction in children’s TSF and higher consumption of ultra-processed foods increased skinfold thickness. These results can be related to parenting style and the obesogenic environment since the number of overweight women (70%) in our study was alarming and we found a high frequency of consumption of processed and ultra-processed foods. The relationship between child feeding and anthropometry is well-defined in the literature and may reflect the consumption of nutrient-poor foods, which are rich in fat and sugars that lead to weight gain [47, 48]. De Araujo et al. (2017) [49] performed a study in Brazil with 548 preschool children aged two to five years and found that the consumption of processed and ultra-processed foods can present the risk of childhood overweight.

Skinfolds are more sensitive to identifying body fat since the two layers of skin are measured along with the subcutaneous fat of a specific point, which can explain the greater impact of food consumption on skinfolds and not on BMI/A [26]. BMI is an adequate marker of the global nutritional index, but it does not distinguish muscle mass from subcutaneous fat (fat mass) and, thus, it is not the best method to identify body fat [50]. Therefore, changes in skinfolds can point to the influence of food on children’s body fat and later affect their BMI and long-term nutritional diagnosis.

This study has some limitations. Firstly, the loss of participants over the period, which, although not unexpected in longitudinal studies [51], introduces bias and compromises power and strong correlations in epidemiological studies. Attrition can be explained by the length of time between enrolment in the two phases of the study and the social circumstances of the participants (including change of address and telephone number). Secondly, we used different tools to assess maternal and children’s food consumption. However, due to the use of three 24hDR questionnaires, we found a frequency of food consumption similar to that of the questionnaire for children, allowing the identification of frequencies of both maternal and children’s food consumption. The use of at least three 24hDRs can help find the frequency of food consumption in the lack of the food frequency questionnaire. Thirdly, we applied the 24hDR on different days of the week, depending on the availability of each mother. Fourth, we did not assess other variables related to the diet quality of parents or guardians to better explain children’s habits and hours of physical exercise and control the effects on the anthropometric profile. Finally, we did not use maternal chronic medical conditions and birth weight as variables of interest in the food consumption and nutritional status of preschoolers, and these are likely to be confounding factors.

This is one of the few studies that analyzed maternal diet quality and classified it according to food groups and levels of processing, showing its influence on the diet quality and nutritional status of children. In the literature, few studies analyze food groups, levels of processing, and anthropometry in preschool children by skinfolds. According to this study, maternal food choices influence their children’s diet quality; however, other factors are related to the food preferences of both. Individual factors are associated, but bigger and more difficult-to-control factors, such as public policies, behavioral patterns, food in schools, and food advertisements aimed at children, should be further studied.

Conducting studies on food consumption is difficult due to the complexity of factors involved in the diet of individuals, which makes data collection more challenging. Besides those aimed at the dietary assessment, other tools should be used to ensure the inclusion of confounding factors. Moreover, food processing data is complex and requires experience and caution. Our research team underwent rigorous training throughout this study, which was essential for its performance.

## Conclusion

Maternal diet quality and the diet and body composition of children are associated. A higher frequency of maternal consumption of unprocessed and/or minimally processed foods was related to a higher frequency of consumption of these foods by children. Moreover, mothers of children with lower SSF values presented a higher frequency of consumption of unprocessed and/or minimally processed foods while mothers of children with excess adiposity by TSF and SSF presented a higher frequency of consumption of ultra-processed foods.

## Supporting information

S1 File Food frequency questionnaire. (DOC)

S1 Data (XLSX)

S1 Table Associations between sociodemographic characteristics and maternal diet quality. SD = standard deviation. p = p-value. MW = minimum wage. 1 MW: R$1.100,00. All tests with the level of significance = 0.05. *Mean ± standard deviation–ANOVA test, post hoc Tukey. **Pearson Correlation. ***Significant correlation.
(DOCX)
