# Supplementary material for: Maternal diet quality and associations with body composition and diet quality of preschool children: A longitudinal study
Source: PLoS One. 2023 May 11;18(5):e0284575. doi: 10.1371/journal.pone.0284575 (PMC10174545; doi:10.1371/journal.pone.0284575)
Supplement: S1 File — (DOC) [file pone.0284575.s001.doc]

| **Questionário de Frequência Alimentar (QFA)** | |
| --- | --- |
| Data da entrevista: __ __ / __ __ / __ __ | **2GDE _ _ / _ _ /_ _** |
| Entrevistador(a): _________________________________________________ | **2ENTREV _____** |
| **Instruções:**  **1. Este questionário avaliará o consumo alimentar de seu(ua) filho(a) durante os últimos 6 meses. Por favor, procure se lembrar dos alimentos que a criança costuma e mais gosta de comer.**  **2. Leia com atenção o nome de cada alimento, a quantidade e as vezes que seu(ua) filho(a) costuma comer o alimento.**  **3. Por favor, marque com um X o quadradinho indicando sempre o consumo mais frequente. Por exemplo, no grupo do *Arroz, pão, massa, batata*, veja se seu filho consome: “Arroz cozido”. Em caso afirmativo, assinale a frequência (número de vezes). Veja o próximo alimento “Batata cozida/purê” e marque da mesma forma. Se a criança não come ou come muito raramente, marque *nunca*.**  **4. Não deixe em branco nenhum dos alimentos do questionário.**  **5. Ao final, existe uma alternativa *Outros*, para o caso de alimentos não citados mas que você habitualmente dá para a criança. Coloque o tipo de alimento, a quantidade e o número de vezes.** | |
