# Supplementary material for: Maternal diet quality and associations with body composition and diet quality of preschool children: A longitudinal study
Source: PLoS One. 2023 May 11;18(5):e0284575. doi: 10.1371/journal.pone.0284575 (PMC10174545; doi:10.1371/journal.pone.0284575)
Supplement: S1 Table — SD = standard deviation. p = p-value. MW = minimum wage. 1 MW: R$1.100,00. All tests with the level of significance = 0.05. *Mean ± standard deviation–ANOVA test, post hoc Tukey. **Pearson Correlation. ***Significant correlation. (DOCX) [file pone.0284575.s003.docx]

**S1 Table – Associations of sociodemographic characteristics with maternal diet quality.**

|  | **Family Income (MW)*** | | | **Maternal education**** | **Maternal age**** |
| --- | --- | --- | --- | --- | --- |
|  | ≤ 1 MW | > 1 a < 3 | ≥ 3 | (years) | (years) |
| **Food Group** | mean±SD | mean±SD | mean±SD | r(p) | r(p) |
| Group 1 | 23.8±4.9 | 22.5±4.7 | 21.7±4.8 | -0.069 (0.536) | -0.111(0.318) |
| Group 2 | 11.8±6.6 | 12.9±7.2 | 17.1±5.1 | 0.216 (0.049)*** | 0.131(0.236) |
| Group 3 | 3.5±37 | 3.8±4.3 | 3.0±3.2 | 0.200 (0.069) | 0.162(0.145) |
| Group 4 | 8.2±4.3 | 8.3±4.2 | 6.4±2.8 | 0.248 (0.024)*** | -0.132(0.234) |
| Group 5 | 13.9±4.9 | 14.1±3.7 | 14.6±3.1 | -0.170 (0.123) | -0.072(0.520) |
| Group 6 | 5.9±3.2 | 6.2±3.2 | 5.0±2.5 | 0.022 (0.843) | 0.053(0.637) |
| Group 7 | 13.0±4.2 | 13.2±3.2 | 14.6±2.3 | -0.341 (0.002)*** | 0.004(0.969) |
| Group 8 | 12.0±6.2 | 12.4±6.1 | 11.8±5.0 | -0.130 (0.240) | 0.080(0.474) |
| Group 9 | 0.3±0.7 | 0.1±0.5 | 0.3±0.7 | 0.176 (0.112) | 0.011(0.925) |
| **Processing Level** |  |  |  |  |  |
| Unprocessed and minimally processed food | 57.2±10,5 | 58.4±11.9 | 60.8±8.5 | -0.108(0.331) | 0.183(0.098) |
| Processed food | 13.2±5.0 | 13.5±5.2 | 9.5±4.2 | 0.060(0.590) | -0.081(0.469) |
| Ultra-processed food | 29.6±10,0 | 28.1±10.7 | 29.7±7.0 | 0.089(0.424) | -0.161(0.145) |

SD= standard deviation. p= p-value. MW= minimum wage. 1 MW: R$1.100,00. All tests with the level of significance =0.05.

*Mean ± standard deviation – ANOVA test, post hoc *Tukey*. **Pearson Correlation. ***Significant correlation.
